# Long-Term Intensive Care Unit (ICU) Stays Can Lead to Long-Term Cognitive Impairment (LTCI): Neurosurgery Nursing Strategies to Minimize Risk

**DOI:** 10.7759/cureus.28967

**Published:** 2022-09-09

**Authors:** Brian Fiani, Ryan Arthur Figueras, Patrick Samones, Claudia Lee, Alexis Castillo, Brian Sangalang, Fatima Garcia Perez

**Affiliations:** 1 Neurosurgery, Weill Cornell Medical Center/NewYork-Presbyterian Hospital, New York, USA; 2 School of Medicine, University of California Riverside, Riverside, USA

**Keywords:** neural processes, cognitive impairment, delirium, dementia, neuro-critical care, nursing strategies, nursing education, intensive care unit, long-term cognitive impairment

## Abstract

Long-term cognitive impairment (LTCI) is a phenomenon predominantly seen in patients within intensive care units (ICU) that causes chronic dysfunction, defined as new or worsening deficits in memory, attention, mental processing speed, executive function, intellectual function, and visual-spatial abilities for over 12 months, inhibiting the necessary return to baseline function without appropriate intervention. Our objective is to provide a guideline of nursing strategies to reduce LTCI through different studies that evaluate pharmacological and non-pharmacological methods. Current literature demonstrates that pharmacotherapy focused on neuronal protection as well as robust physical therapy regimens and regulated sleep schedules show promise in strengthening cognitive function and reducing LTCI. Future studies regarding LTCI should focus on the efficacy of specific pharmacological regimens, large-scale assessments of the implementation of physical therapy to reduce LTCI, as well as, specific interventions to reduce the incidence of delirium in the ICU.

## Introduction and background

The intensive care unit (ICU) is a division of the hospital where patients in critical condition are admitted in order to receive higher levels of care and increased provider supervision until they are stable enough for the medicine floor. A minimum of five million patients are admitted to the ICU in the United States every year, and unfortunately, many patients do not leave the ICU in the same physical or mental state that precedes their illness [1]. In particular, long-term cognitive impairment (LTCI) is highly prevalent in ICU patients over 65 years old, constituting roughly 42% to 52% of ICU patients [2,3]. It is estimated that half of all ICU patients will develop LTCI, defined as new or worsening deficits in memory, attention, mental processing speed, executive function, intellectual function, and visual-spatial abilities, that last greater than twelve months [2,4-6]. Such deficits can impact daily life by affecting patients’ ability to complete instrumental activities of daily living in 60% of patients by causing persistent cognitive impairment that persists for many years after their ICU admission with some studies reporting impairment out to the eight-year mark [5].

The severity of cognitive impairment is dependent on the overall severity of the patient’s skill set impairments. Mild cognitive impairment (MCI) is defined as the impairment in one or more of the aforementioned skill sets though not to the extent that the patient's normal functioning is compromised [7]. MCI is typically age-related while LTCI develops quickly after critical illness in ICU survivors [6]. In a study comparing age and LTCI, ICU patients were split into two groups; the first comprised patients aged 49 and under, and the second comprised patients aged 50 and over. Rates of LTCI grew for both populations; however, the outcomes of cognitive impairment were markedly more severe in the older sample [2].

A condition of severe LTCI is analogous to dementia and mild-to-moderate Alzheimer’s disease (AD), which prevents patients from returning to their baseline cognitive ability [8]. Another form of cognitive impairment is post-operative cognitive impairment, which is cognitive impairment observed after major surgeries, potentially due to anesthesia or hypoxia [9]. It is crucial that interventions targeting a reduction of LTCI rates employ an inter-professional approach; each member of the healthcare team cannot function independently. To date, there has been scant progress in elucidating practical tools for nursing staff to implement, and therefore, the purpose of this investigation is aimed at identifying strategies that nurses in neurosurgical intensive care units may implement to reduce the rates of LTCI in patients.

## Review

Risk factors

In order to prevent cases of LTCI for ICU survivors, it is necessary to understand what factors may contribute to its development. Those factors can generally be categorized into two areas: pre-existing chronic conditions and treatment course. 

Pre-existing Conditions

Delirium is a mental state characterized by inattentiveness, confusion, jumbled thoughts, and oscillating mentation [10]. It may present in up to 80% of ICU patients and has been correlated with greater rates of cognitive impairment [10, 11]. Increasing duration of delirium has also been associated with worsening LTCI [2, 12]. There have not been studies investigating the direct relationship between pre-existing cognitive impairment and the development of LTCI after ICU admission. It has been observed that 48% of ICU patients are above the age of 65 and that dementia is the most common cause of cognitive impairment in the elderly [13,14]. In an outpatient community sample, up to 18.8% of people aged 65 and over had dementia [3]. It was also found that 35% of patients admitted to the ICU had pre-existing cognitive impairments which may worsen during their admission [15]. Thus, the direct relationship between pre-existing cognitive impairment and the development of LTCI after ICU admission remains unclear and is a subject that requires further research.

Miscellaneous chronic medical conditions have also been investigated for potential relationships to the development of LTCI. Cerebrovascular accident, cancer, liver cirrhosis, kidney disease, diabetes, myocardial infarction, peripheral vascular disease, congestive heart failure, chronic pulmonary disease (not interstitial lung disease), and asthma are all conditions that have been associated with increased odds of developing an LTCI [16]. In another study, Sakusic et al. hypothesized that cerebral hypoperfusion and recurrent episodes of severe hypotension, defined as a mean arterial pressure (MAP) < 50 mmHg, could be causative. Another potential cause was prolonged durations of hypoxemia, defined as saturation of peripheral oxygen (SpO2) < 90%, as this also leads to a higher chance of LTCI [16]. Notably, malignancy has been identified as a risk factor for LTCI [17]. In patients with both Type 1 and Type 2 diabetes mellitus, chronic hyperglycemia contributed to cognitive dysfunction [18]. In the outpatient setting, patients with diabetes mellitus that were between the ages of 60-70 years old had a clear association with an increased risk of LTCI and dementia [19].

Medications

Patients that were given quinolones and vancomycin had a strong association with developing post-ICU LTCI [16]. Fluoroquinolone can cause CNS side effects including psychosis, hallucinations, and convulsions, and it is postulated that these neurologic deficits are what may predispose patients to LTCI [20]. 

Mechanical Ventilation

In a small sample study of 77 mechanically ventilated ICU patients, 70% of them were found to have developed LTCI [21]. There have not been studies done on a large scale and in fact, many other studies do not suggest an association between mechanical ventilation and LTCI [22]. Duration of mechanical ventilation is associated with higher odds of LTCI though not statistically significant [16]. The duration of sedation was not found to be correlated to an increased chance of LTCI; however, it was found to increase the rates of delirium which may lead to LTCI [2,22]. Benzodiazepines were not noted to be associated with LTCI but were related to cognitive impairment up to three months after ICU survivorship; thus, more studies need to be conducted in order to evaluate the relationship between sedation and LTCI [2,22,23]. There was no clear relation between propofol, dexmedetomidine, and opiates on post-ICU cognitive impairment [2]; however, some studies suggest that propofol and dexmedetomidine may lead to less severe cognitive impairments [23].

Unnecessary Length of Stay (LOS)

There is no clear relation between the LOS and the development of LTCI. One study noted that an increased LOS in the ICU increases the odds of developing LTCI though not statistically significantly [16]. Another reported that an ICU LOS greater than 27.4 days was linked to a 2.7 times greater chance of cognitive impairment [24].

Clinical trials and outcomes

Clinical trials and studies to date which aim to predict an outcome of LTCI have been focused primarily on delirium as an identifiable primary predictor for LTCI outcomes. The presence of delirium is a predictor of worse cognitive performance in patients at the time of discharge [12,25]. Additionally, an increase in the duration of a delirious state is a positive predictor of worsening cognitive performance, after adjusting for age, education, pre-existing cognitive function, severity of illness, severity of sepsis, and use of sedative medications in the ICU setting [12].

Another study involving 275 patients who were consecutively mechanically ventilated in both medical and coronary ICUs determined that patients who developed delirium throughout their hospital course were associated with higher six-month mortality rates, higher LOS, fewer median days alive, fewer days without mechanical ventilation, and a higher incidence of cognitive impairment at the time of discharge [11]. A longer duration of delirium is associated with poor global cognition in patients who experienced respiratory failure or shock during their ICU course. Thus, in summary, the findings of these studies are highly suggestive of a strong positive correlation between delirium and LTCI. This all highlights an important area for future research [2].

Sleep is known to be important for regulatory neural processes, such as memory consolidation, making the association between sleep and delirium in ICUs an obvious choice. Along with the severity of illness, and physical restraints, sleep deprivation was shown to be an independent predictor of delirium [26]. Additionally, trials comparing traumatic brain injury patients with obstructive sleep apnea (OSA) to those without OSA, of similar age, education, injury severity, and Glasgow Coma Scale scores concluded that patients with OSA performed significantly worse on verbal and visual delayed-recall measures, but both groups performed similarly in motor function tests [27]. 

Another factor of concern when assessing risk for LTCI is mechanical ventilation. Mitchell et al. studied the association of delirium, duration of mechanical ventilation, and LTCI in ICU patients. Both medical and surgical adult patients who had received > 12 hours of mechanical ventilation were assessed for delirium. Ninety-one percent of enrolled patients completed the assessment, incidence of delirium was 19% with 41% cognitively impaired at three months and 24% remaining impaired at six months. At six months, impaired cognition in enrollees with delirium was significant, showing slower processing and executive function speeds compared to those with no delirium. However, in another study assessing delirium daily in mechanically ventilated ICU patients, results showed mechanical ventilation was not independently associated with long-term cognitive impairment [12]. Although mechanical ventilation poses a risk of increased inflammation and muscle atrophy, it needs further investigation to understand the effects of mechanical ventilation on LTCI [21]. 

Nursing strategies

Nurses are well-suited for helping to implement some of the most important interventions for preventing the development of delirium, as they have a lower census of patients each individual is responsible for in the ICU setting, and thus are able to devote a greater level of attention due to lower patient-to-nurse ratios in the ICU setting. Interventions have previously been established which affect cognitive impairment & these have been proposed to delay progression, and even reverse the effects of cognitive impairments. These interventions can be divided into medications, physical training, cognitive interventions, and environmental modifications.

Regarding medications, cerebral enhancing agents such as cholinesterase inhibitors are theorized to reverse pathologic changes in neuronal cells. Because many neurocognitive diseases are associated with a loss of cholinergic neurons, a cholinesterase inhibitor is thought to improve cognitive function by counteracting this deficit. Other cerebral protective agents such as antioxidants and omega-3 fatty acids have beneficial effects by increasing neurotransmitters, enhancing cerebral blood flow, and halting pathological processes; however, there is insufficient data to assert that these beneficial effects will necessarily affect long-term cognitive impairment in either onset or progression [28].

Moderate-intensity physical training has beneficial effects on the cognitive functions of executive function, memory, attention, and overall, improves cognition. Though there is wide variability in the level of exertion and type of physical training intervention, standardization of this form of intervention may very well be a beneficial area of research and investigation in future studies. By merit of the theory of neuroplasticity, there have also been approaches toward applying cognitive interventions to improve cognitive ability in a wide range of patient populations. In particular, there are two implementations that are of note: processing efficiency training, which seeks to achieve improved speeds of processing and dual tasks, and cognitive strategy teaching, which targets higher-order cognitive ability [29,30]. This is particularly helpful in older adults who require compensation for the loss of higher-order cognitive ability. Both approaches demonstrated positive targeted training effects in older adults with cognitive symptoms; however, further studies must delineate any potential benefit in our target patient population of interest, that is, patients who have established LTCI post-ICU admission.

In the discussion of non-pharmacological interventions, sleep hygiene is another intervention that is certainly under-accounted for. In recent observations, Huang et al. found that patients with less disrupted sleep have lower cerebrospinal fluid (CSF) beta-amyloid concentrations, a CSF profile that is related to AD, and have established an association with amnestic cognitive impairment with AD [31]. Thus, it is plausible that the sleep metrics and activity cycles that are deranged are likely associated with AD. Bright-light therapy synchronized with typical daylight patterns, family participation in care, and psychoeducational programs are the three primary single-component interventions that nurses can assist within their daily responsibilities of patient care that have been evaluated in the ICU and associated with decreased risk of development of delirium [32].

Future directions

Based on the clinical trials studied previously, we can identify areas for further research approaches to mitigate the incidence of LTCI. One important area of future study is the effect of patients experiencing delirium and its effect on the patient’s long-term cognitive impairment outcome, as well as their mortality rate following ICU discharge. If delirium can be established to be an influencing factor on post-discharge mortality rates and long-term cognitive impairment outcomes, further studies can follow suit which seek to investigate the causes of delirium and explore effective interventions in the prevention of delirium. Based on this association, assuming it can be established, further studies can be conducted on delirium prevention and its effect on patient mortality and long-term outcomes.

## Conclusions

LTCI is defined as new or worsening deficits in memory, attention, mental processing speed, executive function, intellectual function, and visual-spatial abilities that last greater than 12 months. LTCI can be attributed to pre-existing conditions and treatment course. There are nursing tactics to mitigate the risk of LTCI in the neurosurgical ICU that have already shown success and should be brought into use.
